# US Virgin Islands Launches Modernized NBS 7 Disease Surveillance System to Transform Public Health: Implementation Report

**DOI:** 10.2196/85365

**Published:** 2026-06-23

**Authors:** Hannah M Cranford, Terri Pietka, Leah de Wilde, Marlon Lawrence, Lisa L Ekpo, Esther M Ellis

**Affiliations:** 1 Epidemiology Division, United States Virgin Islands Department of Health, Charles Harwood Complex, 3500 Estate Richmond, Christiansted, United States Virgin Islands, 00820, United States, 1 340 514 7583; 2 Centers for Disease Control and Prevention Foundation, Atlanta, GA, United States; 3 Public Health Laboratory, United States Virgin Islands Department of Health, United States Virgin Islands, United States

**Keywords:** public health surveillance, database, informatics, cloud storage, electronic data processing, public reporting of healthcare data

## Abstract

**Background:**

During January 2024, the US Virgin Islands (USVI) Department of Health (VIDOH) identified a critical need to maintain the cloud-hosted National Electronic Disease Surveillance System Base System (NBS) instance and support the local data modernization initiative. After consulting with federal partners and subject matter experts, VIDOH’s leadership chose to migrate the integrated disease surveillance system to a new platform hosted on Amazon Web Services (AWS) and update the NBS instance to the most advanced version, NBS 7.

**Objective:**

The primary aim was to support a USVI disease surveillance system that is modern, functional, and cost-efficient by migrating the VIDOH NBS instance from a vendor-managed environment to a jurisdiction-managed AWS cloud-based infrastructure while upgrading to NBS 7.

**Methods:**

The VIDOH implemented a phased migration strategy that included planning and cost-benefit assessment, deployment of NBS 7 within AWS, database migration, validation and optimization, and staged reonboarding of electronic reporting facilities.

**Implementation (Results):**

The USVI NBS 7 instance went live on May 6, 2025, with USVI becoming the first US jurisdiction using AWS for implementation of NBS 7 and the second using NBS 7 in production, overall. Benefits of this change included nearly 90% cost savings (preliminarily estimated at 80%), additional bandwidth, real-time data ingestion and updates, an opportunity to build local informatics capacity, and the ability to have greater autonomy over the data and its end points. To date, the VIDOH successfully reonboarded 106 of 109 (97%) previously connected electronic reporting facilities and onboarded 1 new reporting laboratory previously unable to connect due to interoperability barriers.

**Conclusions:**

Updating the USVI database to NBS 7 in a locally owned, cloud-hosted, AWS environment has improved disease surveillance specifically by providing the most up-to-date Centers for Disease Control and Prevention–supported data system, improving timeliness of reporting by offering local providers more flexibility in electronic reporting options, and giving USVI direct control over workflow decision functionality. Furthermore, improved interoperability and maintaining a cloud-based platform were additional benefits of the database migration. This important investment in public health infrastructure will allow USVI public health professionals, clinicians, policymakers, and other stakeholders to be able to monitor and respond to disease threats quickly and inform appropriate public health action.

## Introduction

### Background

During January 2024, the US Virgin Islands (USVI) Department of Health (VIDOH) identified a critical need to maintain its cloud-hosted disease surveillance system and database to support the local public health data modernization initiative. USVI is a US territory consisting of 3 major Caribbean islands, hosting nearly 100,000 residents [1] and an average of 2 million tourists per year [2].

### National Electronic Disease Surveillance System Base System

To protect local public health, the VIDOH uses disease surveillance and investigation to identify, track, and control the spread of reportable diseases and conditions. USVI public health epidemiologists and disease investigators recognize indicators of potential public health threats, monitor disease trends, and document outbreaks via the local National Electronic Disease Surveillance System Base System (NBS) instance [3,4]. NBS is an information system developed by the Centers for Disease Control and Prevention (CDC) that documents disease diagnosis reports from health care providers and laboratories, integrates data from multiple sources, helps health departments manage workflows including case investigation, and transmits disease data to the CDC via electronic notification for national-level reporting and monitoring [4]. The CDC released the modernized NBS version 7.0.0 on October 3, 2023, bringing several new features, enhancing the system responsiveness and adaptability to evolving public health needs and challenges [5]. The modernized NBS is cloud agnostic, supporting Amazon Web Services (AWS), Microsoft Azure, and Google Cloud Platform as runtime options [6]. The platform also adopts a container-based microservices architecture, improving scalability, portability, deployment consistency, and operational flexibility across cloud environments.

The VIDOH has 20 active users of the NBS platform who collect disease reports and document case investigations. During 2024, the USVI NBS instance had Secure File Transfer Protocol connections to 109 reporting facilities sending electronic laboratory reporting (ELR) data. The VIDOH receives an average of 5000 electronic laboratory reports annually, resulting in around 3000 investigations. The VIDOH surveillance system turns raw data into actionable information, allowing local health authorities to protect the USVI population more effectively.

### CDC’s Data Modernization Initiative

During 2019, the CDC launched the Data Modernization Initiative (DMI) to advance disease surveillance systems and data analytics platforms across federal, state, local, and territorial public health systems [7,8]. This nationwide effort focuses on enhancing data quality, interoperability, and real-time sharing to strengthen public health surveillance, response, and decision-making [9]. In alignment with this strategy, the VIDOH has implemented solutions to improve data exchange, automate processes, enhance interoperability, and expand data visualization capabilities to better inform public health actions [10].

### Similar Interventions

To mitigate the loss of on-premises informatics infrastructure during natural disasters such as hurricanes, during 2014, the VIDOH implemented a cloud-hosted NBS environment held and managed by an external informatics vendor. By 2024, the VIDOH NBS instance was on version 6.0.16.1. However, during 2024, increases in vendor pricing and reductions in federal funding to support the VIDOH surveillance system combined to strain local capacity for maintenance.

### Approach

To preserve the USVI NBS instance and support data modernization activities, the VIDOH migrated its disease surveillance database to an AWS cloud platform owned and managed by the jurisdiction and updated the system to the most advanced version, NBS 7. The USVI is the first US jurisdiction using AWS for implementation of NBS 7 and the second US jurisdiction using NBS 7 in production, overall. The aim of this paper is to provide a model for the successful deployment, validation, and configuration of NBS 7 on an AWS cloud infrastructure while confirming operational readiness and adherence to security, performance, and reporting standards.

### Aims and Objectives

The primary objective of this implementation was to migrate the VIDOH NBS instance from a vendor-managed environment to a jurisdiction-managed AWS cloud-based infrastructure while upgrading to NBS 7. Key goals included improving system flexibility, reducing operational costs, increasing local informatics capacity, and expanding the ability to onboard and support ELR from local facilities.

Given the operational nature of this implementation, formal outcome evaluation was not conducted. Instead, progress was assessed using operational indicators, including successful system deployment, continuity of surveillance data following migration, reonboarding of reporting facilities, ELR processing functionality, and system availability. This digital health implementation follows the i-CHECK DH (Guidelines and Checklist for the Reporting on Digital Health Implementations guidelines; Checklist 1) [11].

## Methods

### Implementation Blueprint

The VIDOH implemented a phased migration strategy that included planning and cost-benefit assessment, deployment of NBS 7 within AWS, migration of the legacy database using a backup-and-restore approach, and staged reonboarding of electronic reporting facilities. The implementation emphasized minimizing disruption to surveillance operations, maintaining data integrity, and enabling rapid transition to a jurisdiction-managed environment. Ongoing communication with reporting partners and iterative validation of data flows were used to support system stabilization following launch.

### Technical Design

The implemented solution built upon the existing NBS platform with expansion through deployment in a cloud-based environment aligned with national public health modernization efforts [5-7]. This implementation reflects key principles of the CDC’s DMI, which includes scalable infrastructure, standardized data exchange, and increased jurisdictional control of surveillance systems [8]. The system combines containerized application deployment, managed database services, and serverless data processing to support flexible, scalable, and maintainable surveillance operations.

The NBS 7 environment was implemented within AWS using a modular cloud architecture designed to support secure data ingestion, processing, storage, and reporting access for disease surveillance activities. Core components included Amazon Elastic Kubernetes Service for containerized deployment of the NBS application, Amazon Elastic Compute Cloud for supporting infrastructure, Amazon Relational Database Service for the surveillance database, Amazon Simple Storage Service (S3) for file-based storage and intermediate processing, and AWS Lambda for serverless processing of incoming laboratory data. The system was deployed within a virtual private cloud to isolate resources and control network access. AWS was selected because it was already used by the VIDOH, allowed robust regulations for HIPAA (Health Insurance Portability and Accountability Act) compliance [12], and provided a scalable, flexible infrastructure that allowed the USVI to transition from a vendor-managed model to a jurisdiction-managed environment with greater control over configuration, data pipelines, and ongoing costs.

Electronic laboratory reports are received through secure file transfer and stored in S3, where AWS Lambda scripts were used to parse, transform, and standardize incoming messages into Health Level Seven (HL7)–compliant formats for ingestion into NBS 7. This design supported near real-time processing of incoming reports and provided flexibility to accommodate facilities with varying technical capabilities. For reporting partners without HL7 capacity, the VIDOH also developed a mechanism to ingest comma-separated values flat files and automatically transform them into HL7 formatted messages, improving interoperability and reducing barriers to electronic reporting. The overall data lifecycle includes secure collection of incoming reports, automated processing and transformation, storage within AWS-managed services, and controlled access for surveillance, investigation, and reporting activities.

Access to AWS resources was managed using identity-based and role-based permissions following a least-privilege approach. Permissions were structured to differentiate system development and administration and data processing functions. Sensitive data were protected through encryption in transit and at rest, including encrypted storage for the database and file-based data repositories. Logging and monitoring mechanisms were used to track system activity, data ingestion, message transformation, and processing outcomes, supporting operational monitoring, troubleshooting, and auditability.

Under the previous vendor-hosted NBS 6 instance, access to the NBS application was managed through role-based multifactor authentication. NBS 7 security was maintained with similar role-based features integrated with Keycloak, a system that has native multifactor authentication capabilities. Individuals with access to the NBS interface were assigned permissions based on least-privilege principles, with access restricted to appropriate program areas and user roles to ensure that sensitive data were only available to authorized personnel.

In accordance with AWS’s shared responsibility model, AWS was responsible for the security of the underlying cloud infrastructure, while the VIDOH was responsible for secure configuration of services, access control, and governance of the data hosted within the environment. The system was designed to support secure handling of protected health information and to align with applicable public health data management requirements.

To support continuity of operations, routine snapshots of system components were performed, including automated backups and snapshots of the Amazon Relational Database Service database and supporting infrastructure. S3 provides durable, redundant storage for inbound and processed data files. These measures supported recovery of key system components in the event of failure and reduced the risk of data loss during ongoing surveillance operations. The architecture also improved the VIDOH’s ability to support direct connections, customize data transformations for local facilities, and expand ELR in a way that was more adaptable to local public health needs.

### Target Setting and Population

The implementation targeted the VIDOH surveillance infrastructure and its supporting reporting ecosystem, including public health staff, local laboratories, and other reporting facilities responsible for submitting notifiable disease data. The system was deployed within the USVI and designed to support territorial public health surveillance operations, with particular emphasis on improving ELR capabilities and reducing barriers for facilities with limited technical resources.

### Data Governance

Surveillance data were collected from reporting facilities, processed and standardized through automated workflows, and stored within AWS-managed services. The VIDOH maintained ownership and control of the data, with access restricted to authorized users for public health purposes. Data handling practices were designed to support secure processing of routine surveillance data and align with applicable public health data protection requirements.

### Interoperability

The system was designed to support interoperability through the use of HL7 messaging standards for ELR. Custom data transformation processes enabled integration with a range of laboratory systems, including those without native HL7 capability, by converting alternative data formats into HL7-compliant messages for ingestion into NBS. Via such methods, the VIDOH can consider future integrations and connections with additional local systems and databases such as the Territorial vaccination registry, among others.

### Participating Entities

The implementation was led by the VIDOH in collaboration with the CDC and CDC-contracted technical partners. The VIDOH provided leadership, system ownership, and operational support, while the CDC and partners provided technical guidance, infrastructure support, and knowledge transfer. The system is owned and maintained by the VIDOH following implementation.

### Budget Planning

A cost-benefit analysis was conducted prior to implementation to evaluate the transition from a vendor-hosted model to AWS. The analysis accounted for infrastructure, implementation support, and other resources. The analysis projected future cost savings to be nearly 80%, following the successful implementation of NBS 7 on AWS. This estimate is limited to infrastructure costs and does not include staffing and personnel resources required to support and maintain the system. This estimate also assumes the annual NBS use remains stable and that the VIDOH retains the technical capacity needed to operate and sustain the system over time.

### Sustainability

A sustainability plan was developed focusing on maintaining uninterrupted disease surveillance operations, minimizing infrastructure and staffing risks, and supporting modernization efforts aligned with public health priorities. The plan emphasized financial sustainability through the establishment of diversified funding sources and maintaining reserve funds to mitigate risks associated with governmental procurement delays, plus implementing cloud cost optimization measures to further reduce operational costs (ie, scheduling the test environment to run only during business hours). The plan also highlighted the importance of sustaining a skilled technical workforce through cross-training, succession planning, documentation, and workforce development while leveraging current technical professionals (data scientists and database engineers) and contractors supporting the VIDOH. Additional priorities included maintaining strong security and disaster recovery practices, supporting interoperability and modernization, and establishing governance structures and performance metrics to guide ongoing system maintenance and improvements.

### Ethical Considerations

VIDOH confirms that no ethics approval was required for this report, as it details informatics processes and did not involve human subjects, animal testing, or confidential data. The VIDOH is a legally designated public health authority that allows permissive disclosure of information without authorization under the 2019 U.S. Virgin Islands Code Title 19 [13]. As this system supports routine public health surveillance, patient consent is governed by existing public health reporting regulations.

## Implementation (Results)

### Outcomes

**Figure 1.**
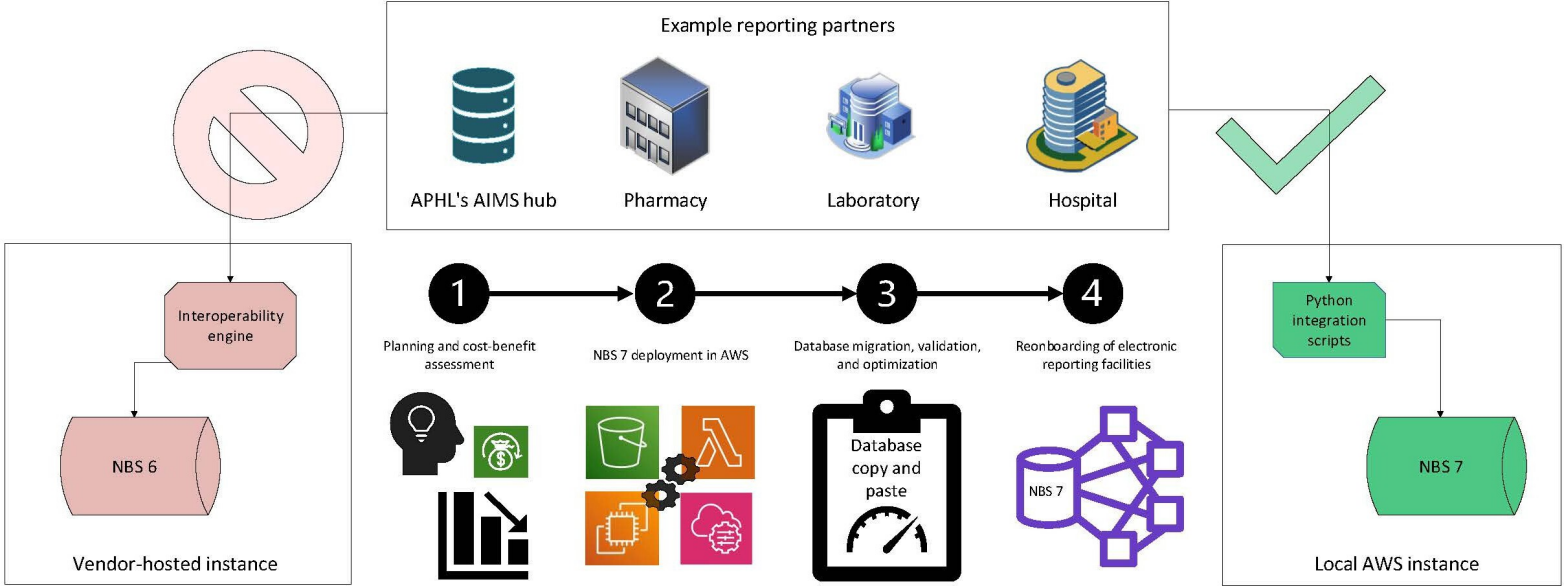
Implementation schema for the US Virgin Islands Department of Health’s National Electronic Disease Surveillance System Base System (NBS) upgrade and cloud-hosted Amazon Web Services (AWS) migration. APHL: Association of Public Health Laboratories.

The USVI NBS 7 instance went live on May 6, 2025, after standing up the new test and production NBS environments on AWS, copying and transferring the existing database, and developing protocols to navigate existing electronic reporting feeds to the new system (Figure 1). Coverage of the NBS 7 platform is Territory-wide, comprehensive of all active users of the platform. A total of 10 months after the initial cutover, the VIDOH informatics team successfully reonboarded 106 out of 109 (97%) previously connected electronic reporting facilities. The remaining 3 previously connected facilities were not reonboarded due to the discontinuation of laboratory testing services or lack of reportable disease testing at their facility. Additionally, the VIDOH conducted onboarding for 1 new reporting laboratory, previously unable to connect due to interoperability barriers. The VIDOH continues facility onboarding for new ELR feeds.

Following an upgrade to NBS 7 and migration to AWS, the VIDOH observed major indicators of success. First, within the first month of operation, infrastructure-related costs were reduced by approximately 85%, exceeding our preliminary estimate of 80%. Further system optimization of system resources has reduced operational costs by approximately 90%. Overall, lower costs were driven primarily by the elimination of vendor hosting and licensing fees and the adoption of a scalable, pay-as-you-go cloud model with local support and maintenance. Second, users experienced enhanced operational performance, increased system bandwidth, and improved application speed. Third, the VIDOH had the opportunity to build local informatics capacity, upskill local employees, and cross-train team members in informatics, data science, and epidemiology. Finally, with increased jurisdictional control over the surveillance system, the VIDOH gained the ability to have greater autonomy over the data and its endpoints. VIDOH staff not only have access to the user interface but can now directly access the database tables and run SQL queries for information previously unavailable or available via a time and materials surcharge from the vendor. Examples of these types of data include lists of active users, outbreak names, and local lab tests with Logical Observation Identifiers Names and Codes or Systematized Nomenclature of Medicine codes.

### Public Health Impact

In rural and limited resource settings, such as the USVI, local hospitals and other reporters face major barriers to electronic disease reporting, including lack of capacity to electronically exchange information, interface-related issues, different vocabulary standards, and difficulty extracting relevant information from the electronic health record [14,15]. NBS requires specifically formatted HL7 messages for successful ingestion. However, many facilities with rigid or outdated electronic health records or laboratory information management systems are unable to generate or adjust messages to the exact specifications required for this database.

As a result of the migration and expanded USVI informatics capacity, the VIDOH is now able to support local facilities in direct electronic reporting, an option that was previously unavailable due to interoperability barriers. To address limitations among facilities with limited information technology resources, the VIDOH built and used AWS Lambda scripts to customize HL7 messaging and support translation and mapping of incoming data that could not otherwise be standardized to HL7 format. Additionally, the VIDOH developed a mechanism to ingest comma-separated value flat files and automatically standardize and transform them into HL7 messages, further promoting equitable electronic reporting participation.

The ability to directly manage onboarding processes and customize data transformations has strengthened relationships with reporting partners and improved the completeness and timeliness of incoming disease reports, supporting more effective public health monitoring and response. Furthermore, these enhancements support the broader goals of the CDC’s DMI by addressing interoperability gaps, simplifying data exchange complexity, promoting equitable public health data exchange, and ensuring timely actionable data [9,10].

### Lessons Learned

While USVI is the second jurisdiction to deploy NBS 7 in a production environment, it is the first to implement it on AWS. NBS 7 was designed to be cloud agnostic (deployable on AWS, Azure, etc), and the VIDOH’s implementation on AWS provides validation of this approach. For jurisdictions transitioning to a cloud-hosted disease surveillance system, NBS 7 offers advantages through its flexible deployment within existing cloud infrastructure or a preferred platform. Key considerations for other jurisdictions exploring cloud-hosting include engaging cloud architects and database engineers for system setup, establishing detailed protocols and documentation, and providing robust training for local staff in cloud technologies, integration engines, programming languages (eg, Python and SQL), and the public health data formatting and coding standards needed to ensure interoperability and sustainability (eg, HL7).

### Challenges and Unintended Consequences

The VIDOH faced multiple challenges and unintended consequences throughout the database transition. First, a large effort was needed by multiple teams from the VIDOH and the CDC to stand up the new environment in AWS. This project brought together many collaborators with distinct subject matter expertise including database engineers, data scientists, information technology specialists, NBS modernization experts, project managers, coordinators, user representatives, and local leaders. Despite the significant effort required, bringing all stakeholders to the table ultimately resulted in a well-designed plan and smooth execution.

Second, there was a data delay resulting from the reonboarding of existing electronic reporting feeds to the new system. However, gaps in ELR submissions were mitigated by requesting reporting facilities to send historical data from the date of cutover up to the date of reboarding. One local reporting facility was unable to send historical data for the 5-month gap; however, the facility is small and has traditionally maintained a low reporting volume.

Third, this project highlighted the limitations of local informatics capacity. Constraints in staffing, technical expertise, and long-term federal support for informatics infrastructure in a resource-limited jurisdiction, such as USVI, can hinder the implementation, maintenance, and optimization of modern databases and create barriers to achieving a fully streamlined and resilient public health disease surveillance system. One unintended, positive outcome of this project was that, through the strengthened partnership developed with the CDC, the VIDOH became a beta testing site for all NBS 7 upgrades, enabling the department to receive more direct, hands-on support from the CDC information technology team moving forward. The VIDOH continues to prioritize sustainability and investment in informatics infrastructure through the acquisition of diverse funding sources, system optimization, strategic stakeholder engagement, staff retention, and ongoing knowledge transfer and cross-training.

## Discussion

### Principal Findings

The VIDOH, with backing from the CDC, launched the USVI’s modernized disease surveillance system, NBS 7, in a jurisdiction-owned and managed AWS environment. This project was cost-effective (approximately 90% cost reduction), used the most up-to-date CDC-supported data system, and delivered improved public health action through increased timeliness of reporting by offering local providers more flexibility in electronic reporting options. Additional benefits of database migration included enhanced interoperability capabilities, maintaining a cloud-based platform, and giving the VIDOH direct control over workflow decision functionality. This increased control over the reporting data pipeline also enables the VIDOH to assess and monitor key surveillance performance indicators more directly, including reporting completeness, timeliness and delays, and facility coverage rates, which were previously more difficult to evaluate in a vendor-managed environment.

### Future Implications

DMI activities continue in USVI with the modernization of both outgoing and incoming data exchange to strengthen the local disease surveillance system. This includes adopting standardized messaging protocols for reporting to the CDC and building the capability to receive and ingest electronic case report data feeds. The VIDOH also aims to develop application programming interfaces to seamlessly share data with the USVI Epidemiology Data Dashboard [16] and other analytic platforms.

### Conclusions

Investing in a modern, functional, and cost-efficient disease surveillance system is essential for the USVI to effectively detect, monitor, and respond to emerging health threats. This report can serve as a practical guide for other jurisdictions working to strengthen the capacity of their own surveillance systems. Enhancing this infrastructure will improve the timeliness and accuracy of public health reporting while fostering stronger collaboration among providers, clinicians, policymakers, and other key stakeholders. By equipping the Territory with reliable, scalable, and modern surveillance tools, this initiative will support evidence-based decision-making and ensure that public health actions remain responsive to community needs.

## Supplementary material

10.2196/85365 Checklist 1 i-CHECK DH guidelines checklist.

## Abbreviations:

AWS: Amazon Web Services
CDC: Centers for Disease Control and Prevention
DMI: Data Modernization Initiative
ELR: electronic laboratory reporting
HIPAA: Health Insurance Portability and Accountability Act
HL7: Health Level Seven
i-CHECK DH: Guidelines and Checklist for the Reporting on Digital Health Implementations guidelines
NBS: National Electronic Disease Surveillance System Base System
S3: Simple Storage Service
USVI: United States Virgin Islands
VIDOH: United States Virgin Islands Department of Health
